# Residual Pulmonary Vascular Resistance Increase Under Left Ventricular Assist Device Support Predicts Long-Term Cardiac Function After Heart Transplantation

**DOI:** 10.3389/fcvm.2022.904350

**Published:** 2022-06-01

**Authors:** Nobutaka Kakuda, Eisuke Amiya, Masaru Hatano, Masaki Tsuji, Chie Bujo, Junichi Ishida, Hiroki Yagi, Akihito Saito, Koichi Narita, Yoshitaka Isotani, Kanna Fujita, Masahiko Ando, Shogo Shimada, Osamu Kinoshita, Minoru Ono, Issei Komuro

**Affiliations:** ^1^Department of Cardiovascular Medicine, Graduate School of Medicine, University of Tokyo, Bunkyo-ku, Japan; ^2^Department of Therapeutic Strategy for Heart Failure, University of Tokyo, Bunkyo-ku, Japan; ^3^Advanced Medical Center for Heart Failure, University of Tokyo, Bunkyo-ku, Japan; ^4^Department of Computational Radiology and Preventive Medicine, The University of Tokyo Hospital, Bunkyo-ku, Japan; ^5^Department of Cardiac Surgery, University of Tokyo, Bunkyo-ku, Japan

**Keywords:** heart transplantation, pulmonary vascular resistance, left ventricular assist device, heart failure, rejection

## Abstract

**Aims:**

We compared hemodynamics and clinical events after heart transplantation (HTx) in patients stratified by the severity of residual pulmonary vascular resistance (PVR) after left ventricular assist device (LVAD) implantation for bridge to transplantation.

**Methods:**

We retrospectively analyzed patients who had undergone HTx at the University of Tokyo Hospital. We defined the high PVR group as patients with PVR of >3 Wood Units (WU) as measured by right heart catheterization performed 1 month after LVAD implantation.

**Results:**

We included 85 consecutive HTx recipients, 20 of whom were classified in the high PVR group and 65 in the low PVR group. The difference in PVR between the two groups became apparent at 2 years after HTx (the high PVR group: 1.77 ± 0.41 WU, the low PVR group: 1.24 ± 0.59 WU, *p* = 0.0009). The differences in mean pulmonary artery pressure (mPAP), mean right arterial pressure (mRAP), and mean pulmonary capillary wedge pressure (mPCWP) tended to increase from the first year after HTx, and were all significantly higher in the high PVR group at 3 years after HTx (mPAP: 22.7 ± 9.0 mm Hg vs. 15.4 ± 4.3 mm Hg, *p* = 0.0009, mRAP: 7.2 ± 3.6 mm Hg vs. 4.1 ± 2.1 mm Hg, *p* = 0.0042, and mPCWP: 13.4 ± 4.5 mm Hg, 8.8 ± 3.3 mm Hg, *p* = 0.0040). In addition, pulmonary artery pulsatility index was significantly lower in the high PVR group than in the low PVR group at 3 years after HTx (2.51 ± 1.00 vs. 5.21 ± 3.23, *p* = 0.0033). The composite event including hospitalization for heart failure, diuretic use, and elevated intracardiac pressure (mRAP ≥ 12 mm Hg or mPCWP ≥ 18 mm Hg) between the two groups was significantly more common in the high PVR group. Residual high PVR was still an important predictor (hazard ratio 6.5, 95% confidence interval 2.0–21.6, and *p* = 0.0023) after multivariate Cox regression analysis.

**Conclusion:**

Our study demonstrates that patients with residual high PVR under LVAD implantation showed the increase of right and left atrial pressure in the chronic phase after HTx.

## Introduction

Heart transplantation (HTx) is a treatment method for severe heart failure in which a patient’s heart, which cannot be saved by conventional treatment methods, is replaced with a healthy heart from a brain-dead person. However, shortage of donor heart is a common worldwide issue that limits the benefit of HTx (1, 2). To overcome the issue about shortage of donor, mechanical circulatory support is available as bridge to transplant (BTT) therapy (3). Indeed, in Japan, most patients require a ventricular assist device as a BTT device that can be used for a long time before HTx. Generally, patients with severe heart failure often have pulmonary hypertension, which is a poor prognostic factor (4). Sustained retrograde propagation of increased left atrial pressure to the pulmonary artery due to left heart failure leads to contraction and remodeling of the pulmonary artery, resulting in increased pulmonary vascular resistance (PVR) and, ultimately, right ventricular dysfunction (5, 6). In patients with heart failure, a left ventricular assist device (LVAD) reduces the load on the left ventricle and PVR (7–9) but long-term burden derived from left-sided heart failure on the pulmonary vascular system is thought to cause irreversible changes in the pulmonary vascular system (6). LVAD support alleviates the burden of left side heart and decrease the left atrial pressure significantly, resulting in improvement of pulmonary hypertension (10). However, some cases in which PVR increase could not be improved sufficiently under LVAD support. Since the recipient’s pulmonary vascular system remains unaffected by HTx, recipient pulmonary vascular degeneration may affect the post-HTx course. There are many unclear points about the impacts of pre-HTx PVR on long-term clinical course after HTx. Therefore, in this study, we investigated the clinical parameters and hemodynamics after HTx in patients stratified by the severity of PVR before HTx, specifically after LVAD implantation.

## Materials and Methods

### Patient Selection

All patients were implanted with LVAD at our institution between October 2004 and September 2018 or visited our institution’s outpatient clinic after undergoing LVAD implantation at another institution during the same period. Patients who underwent right heart catheterization for hemodynamic evaluation 1 month after LVAD implantation and were ultimately able to undergo cardiac transplantation were selected. Patients who died within 30 days of HTx were excluded (*N* = 2). LVAD therapy included HeartMate II (Thoratec Corporation, Pleasanton, United States), EVAHEART (Sun Medical Company, Moriyama, Tokyo, Japan), Jarvik 2000 (Century Medical, Inc., Tokyo, Japan), DuraHeart (TERUMO, Tokyo, Japan), HVAD (Medtronic PLC, Dublin, Ireland), and Nipro-VAD (Nipro Corporation., Osaka, Japan). The study protocol conformed to the tenets of the Declaration of Helsinki, and was reviewed and approved by the University of Tokyo Institutional Review Board (approval number: 2650).

### Procedure of Heart Transplantation

All transplanted hearts were obtained from a brain-dead donor with a beating heart, stored in a cooled cardiac preservation solution, and transported cold. All heart transplants were performed using standard procedures with bicaval anastomoses. All patients who underwent transplantation were treated with standard immunosuppressive therapy using three drugs: calcineurin inhibitor, mycophenolate mofetil, and prednisolone. In the middle of the course, there were some cases in which mycophenolate mofetil was changed to everolimus. The doses of calcineurin inhibitor and everolimus were strictly controlled by adjusting the trough concentration according to the number of weeks after transplantation. Prednisolone administration was decreased over time and discontinued within a year. When mild acute cellular rejection (ACR; grade ≤ 1R) was present, only optimization of immunosuppression was performed, but when moderate ACR [grade ≥ 2R, which means that two or more foci of mononuclear cells (lymphocytes/macrophages) with associated myocyte damage are present (11, 12)] was present, optimization of immunosuppression and pulse steroids was performed. When antibody-mediated rejection was diagnosed, optimization of maintenance immunosuppression, intravenous gamma globulin, pulse steroids, or plasmapheresis was considered.

### Cardiac Catheterization

One month after LVAD implantation, right heart catheterization was performed for hemodynamic evaluation. Conversely, after HTx, right heart catheterization and endomyocardial biopsy from the right ventricular middle septum were regularly performed 1, 2, 3, 4, 6, 8, 12, 18, and 24 weeks according to the institutional protocol. It was performed yearly, 1 year after HTx. In this study, we used right heart catheterization data 1 month after LVAD implantation and 24 weeks, 1 year, 2 years, and 3 years after HTx. Right heart catheterization was performed by inserting a Swan Ganz catheter through the jugular or femoral vein. Right atrial pressure (RAP), right ventricular pressure, pulmonary artery pressure (PAP), pulmonary artery wedge pressure (PCWP), cardiac output (CO), and cardiac index (CI) were measured by Fick’s principle. In this study, we used mean right atrioventricular pressure (mRAP), mean pulmonary artery pressure (mPAP), mean pulmonary artery wedge pressure (mPCWP), and CI. The PVR, pulmonary artery pulsatility index (PAPi), diastolic pressure gradient (DPG), and transpulmonary gradient (TPG) were calculated as follows; PVR, (mPAP-mPCWP)/CO; PAPi, (sPAP-dPAP)/mRAP; DPG, dPAP-mPCWP; and TPG, mPAP-mPCWP. On the LVAD support, the setting of LVAD speed was generally set with reference of clinical findings and echocardiographic findings before right heart catheterization. In the right heart catheterization, hemodynamic data and echocardiographic measurements were recorded at the patient’s initial baseline LVAD speed. The LVAD speed was then lowered to several speeds with repeat collection of hemodynamic and echocardiographic data. The LVAD speed was then increased to several speeds with repeat data collection at each interval. We got the data of hemodynamics at the first LVAD speed in right heart catheterization. The right heart catheterization was identically performed for different LVADs.

### Blood Test

Blood tests were performed 1 month after LVAD implantation and sequentially after HTx. In this study, we collected data at 1 month after LVAD implantation and 4 weeks, 24 weeks, 1 year, and 3 years after HTx.

### Echocardiography

Echocardiography was performed sequentially after HTx. Normal echocardiographic parameters, including left ventricular diastolic dimension (LVDs), left ventricular dimension (LVDd), left ventricular ejection fraction (LVEF), and E/e’, were evaluated. The *e*’ value is the average of the values measured at both the septum and the lateral wall. We collected the data 1 week, 24 weeks, 1 year, and 3 years after HTx.

### Endomyocardial Biopsy

Using the diagnostic criteria for ACR by the International Society for Heart and Lung Transplantation (ISHLT), cellular rejection of grade 2R (ISHLT2004)/3A (ISHLT1990), or higher was classified as positive ACR.

### Evaluated Variables

Right ventricular failure (RVF) was defined and classified according to the International Mechanically Assisted Circulatory Support (INTERMACS) Registry, in which late RVF was defined as meeting the moderate or severe INTERMACS RVF definition after discharge from the index LVAD implant, presence of inotrope, use of intravenous or inhaled pulmonary vasodilators for a duration of >1 week, or needing RV assist device implantation (13).

Donor-recipient sex mismatch was defined as a female donor to a male recipient (14). Donor-recipient body weight mismatch was defined as the ratio of donor-to-recipient weight to <−20% or >30% (12).

In this study, using the general definition of pulmonary hypertension (15), cases with PVR > 3 Wood units (WU) on right heart catheterization 1 month after LVAD implantation were classified as the high PVR group and others were defined as the low PVR group.

During follow-up after HTx, we defined worsening heart failure as the composite including heart failure hospitalization, diuretic use for the treatment of congestion, and elevated intracardiac pressure (16).

### Statistical Analysis

Data were expressed as mean ± standard deviation or median (interquartile range). Statistical analysis was performed using JMP software (version 14.2; SAS Institute, Cary, NC, United States). Student’s *t*-test or Mann–Whitney *U* test was used for continuous variables, while Fisher’s exact test was used for categorical variables. Right heart catheter and blood data from a baseline of 1 month after LVAD implantation to each period after HTx and echocardiography data from the harvest to each period after HTx were analyzed using the paired *t*-test or Wilcoxon signed-rank test. The Bonferroni method was used to assess the significance of the multiple comparisons. To examine changes over time in PVR, PAPi, mRAP, mPAP, mPCWP, and CI, the mean value of each parameter was graphed in patients stratified by baseline PVR. Kaplan–Meier survival analysis was performed to assess (1) survival after HTx; (2) hospitalization for heart failure; and (3) composite events of hospitalization for heart failure, diuretic use for the treatment of congestion, and elevated intracardiac pressure (mRAP ≥ 12 mm Hg or mPCWP ≥ 18 mm Hg) between the two groups, which were compared using a log-rank test. Cox regression analysis was performed to explore significant predictors of the composite events of heart failure hospitalization, diuretic use for the treatment of congestion, and elevated intracardiac pressure. The cutoff value of each variable, except BMI and eGFR, for the hazard analysis was calculated using a receiver operating characteristic curve. We selected values that maximized the sum of sensitivity and specificity as the cutoff values to calculate the area under the curve. Multivariate analysis was performed using variables for which *P* < 0.1 was obtained by univariate analysis. Statistical significance was set at a *P*-value < 0.05.

## Results

### Baseline Patient and Transplanted Heart Characteristics

**FIGURE 1:**
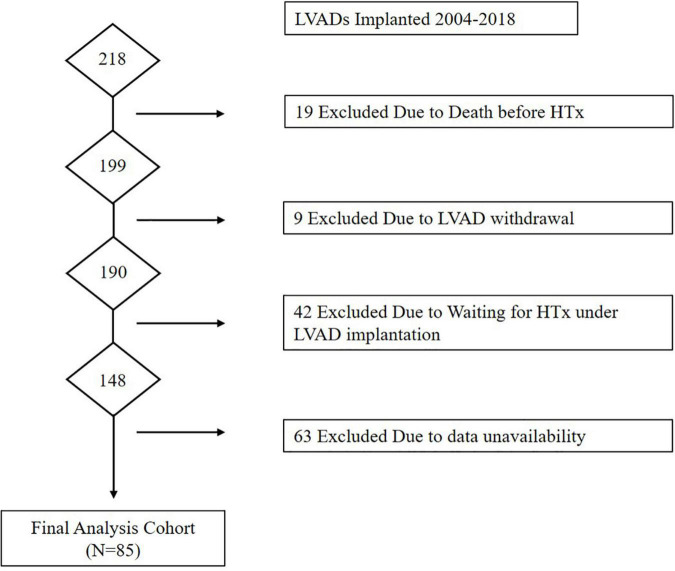
Flow of patients through the Study. HTx, heart transplantation; LVAD, left ventricular assist device.

Flow of patients through the study is presented in Figure 1. A total of 218 patients were implanted with LVAD during the study period. Of these, 19 were excluded due to death before HTx, 9 were excluded due to removal of LVAD due to functional recovery, 42 were excluded because they were still awaiting HTx under LVAD, and 63 were excluded due to absence of right heart catheter data at 1 month after LVAD implantation. A total of 85 patients were eligible for the final analysis of which 20 patients were classified in the high PVR group and 65 in the low PVR group. Table 1 shows the baseline characteristics (which was measured on LVAD support) of two groups. Age at HTx was significantly older in the high PVR group (high PVR group, 45.7 ± 12.0 years; low PVR group, 39.0 ± 13.1 years; *p* = 0.043). BMI was also significantly higher in the high PVR group (high PVR group, 23.2 ± 3.9; low PVR group, 21.5 ± 3.1; *p* = 0.046). According to the types of LVAD, 29 patients were implanted with HeartMate II, 20 patients with EVAHERAT, 16 patients with Jarvik 2000, 13 patients with DuraHeart, 6 patients with Nipro-LVAD, and 1 patient with HVAD. No significant differences were found in the LVAD model between the two groups. There was no significant difference in the presence of significant tricuspid regurgitation. During LVAD support, RVF was observed in 12 patients (14.1%), which was not associated with the presence of high PVR. Table 2 shows the clinical characteristics in the donors and relationship between donors and recipients in the two groups. There was no significant difference in basic donor and echocardiographic parameters at the time of harvest between the two groups. Donor-to-recipient body weight mismatch was significantly higher in the low PVR group.

**TABLE 1:** Baseline demographic and clinical parameters of recipients in two groups.

	High PVR (*n* = 20)	Low PVR (*n* = 65)	*P* value
**Recipient factors**			
Age at HTx, years	45.7 ± 12.0	38.9 ± 13.1	0.043*
BMI	23.2 ± 3.9	21.5 ± 3.1	0.046*
Body surface area, m^2^	1.66 ± 0.16	1.68 ± 0.19	0.64
Male, *n* (%)	12 (60)	48 (73.9)	0.27
**Primary disease**			
DCM, *n* (%)	11 (55)	49 (75)	0.10
D-HCM, *n* (%)	4 (20)	8 (12)	0.47
ICM, *n* (%)	2 (10)	5 (7.7)	0.67
ARVC, *n* (%)	0	2 (3)	–
Cardiac sarcoidosis, *n* (%)	1 (5)	1 (2)	0.42
LVNC, *n* (%)	1 (5)	0	0.24
Drug-induced cardiomyopathy, *n* (%)	1 (5)	0	0.24
**History of RVF while LVAD treatment, *n* (%)**	2 (10)	10 (15.4)	0.72
**Types of LVAD, *n* (%)**			
HeartMate II	7 (35)	22 (33.9)	0.92
EVAHEART	4 (20)	16 (24.6)	0.77
Jarvik2000	6 (30)	10 (15.4)	0.19
DuraHeart	1 (5)	12 (18.5)	0.28
Nipro-VAD	2 (10)	4 (6.2)	0.62
HVAD	0 (0)	1 (1.5)	–
**Days from LVAD implantation to HTx, days**	1,330 ± 420	1,200 ± 450	0.26
**Days from onset of cardiac disease to the date of catheterization after LVAD implantation, days**	3,188 (1,303, 5,413)	1,771 (658, 3,665)	0.11
**Blood test 1 month after LVAD implantation**			
BNP, pg/ml	313.8 (173.3, 682.5)	195.5 (155, 495.2)	0.23
Alb, g/dl	3.3 (3, 3.6)	3.3 (3.1, 3.7)	0.48
Hb, g/dl	10.2 (9.4, 11.7)	10.5 (9.8, 11.5)	0.33
eGFR, ml/min/1.73 m^2^	88.9 ± 31.4	94.6 ± 37.3	0.56

PVR, pulmonary vascular resistance; HTx, heart transplantation; BMI, body mass index; DCM, dilated cardiomyopathy; D-HCM, dilated phase of hypertrophic cardiomyopathy; ICM, ischemic cardiomyopathy; ARVC, arrhythmogenic right ventricular cardiomyopathy; LVNC, left ventricular non-compaction; RVF, right ventricular failure; LVAD, left ventricular assist device; BNP, brain natriuretic peptide; and eGFR, estimated glomerular filtration rate. *p < 0.05.

**TABLE 2:** Baseline demographic and clinical parameters of donors, clinical course after HTx and donor-recipient relationship factors in two groups.

	High PVR (*n* = 20)	Low PVR (*n* = 65)	*P* value
**Donor factors**			
Age, years	48.4 ± 10.4	43.2 ± 14.8	0.25
BMI	22.7 ± 2.6	23.0 ± 4.5	0.74
Body surface area, m^2^	1.69 ± 0.18	1.68 ± 0.22	0.85
Male, *n* (%)	9 (45)	39 (60)	0.30
Cardiac arrest, *n* (%)	8/17 (47)	30/59 (51)	0.78
Cerebrovascular cause for brain death, *n* (%)	11 (55)	29 (45)	0.45
ST-T change of Electrocardiogram, *n* (%)	4 (24)	16 (25)	0.87
LVDd, mm	42.3 ± 5.4	44.7 ± 5.7	0.14
LVDs, mm	28.8 ± 5.4	29.4 ± 4.4	0.65
LVEF,%	60.9 ± 9.8	62.3 ± 7.0	0.49
**Perioperative factors**			
Total ischemic time, min	281 (227, 296)	245 (225, 264)	0.082
**Donor-recipient relationship factors**			
Sex mismatch, *n* (%)	4 (20)	12 (18)	0.88
Donor to recipient BW mismatch < −20% or >30%, *n* (%)	0 (0)	16 (25)	0.017*

PVR, pulmonary vascular resistance; BMI, body mass index; LVDd, left ventricular end-diastolic diameter; LVDs, left ventricular end-systolic diameter; LVEF, left ventricular ejection fraction; and BW, body weight. *p < 0.05.

### Acute Cellular Rejection After Heart Transplantation

Table 3 shows the incidence of ACR within and after 1 year post-HTx in both groups. There was no significant difference in ACR between the two groups in either the acute or chronic phase.

**TABLE 3:** Acute cellular rejection and CAV in two groups.

	High PVR	Low PVR	*P* value
**Acute cellular rejection (>grade2R)**			
within 6 months after HTx, *n* (%)	11/20 (55)	34/65 (52)	0.83
Between 1 and 3 years after HTx, *n* (%)	4/14 (29)	9/60 (15)	0.25
**CAV at 1 year after HTx**			
ISHLT CAV_1–3_, *n* (%)	7/17 (41)	14/51 (27)	0.37
**CAV at 2 years after HTx**			
ISHLT CAV_1–3_, *n* (%)	7/11 (64)	12/39 (31)	0.08
**CAV at 3 years after HTx**			
ISHLT CAV_1–3_, *n* (%)	6/11 (55)	13/34 (38)	0.49

*PVR, pulmonary vascular resistance; HTx, heart transplantation; CAV, cardiac allograft vasculopathy; and ISHLT, International Society for Heart and Lung Transplantation.*

### Change of Clinical Parameters After Heart Transplantation

**FIGURE 2:**
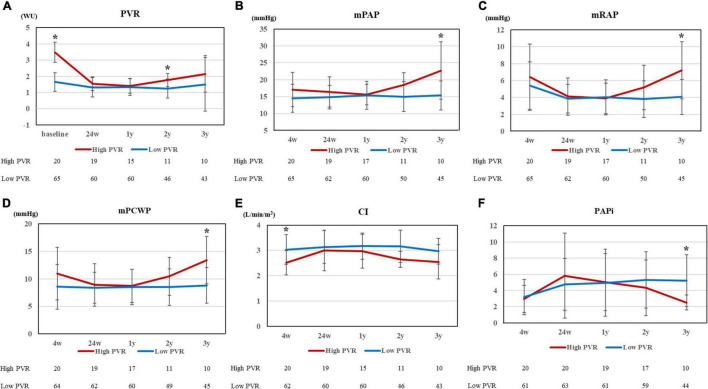
**(A)** PVR at 5 points (baseline, 24 weeks, 1 year, 2 years, and 3 years after HTx), **(B)** mPAP, **(C)** mRAP, **(D)** mPCWP, **(E)** CI, and **(F)** PAPi at 5 points (4 weeks, 24 weeks, 1 year, 2 years, and 3 years after HTx) for high and low PVR group. PVR, pulmonary vascular resistance; HTx, heart transplantation; mPAP, mean pulmonary artery pressure; mRAP, mean right arterial pressure; mPCWP, mean pulmonary capillary wedge pressure; CI, cardiac index; and PAPi, pulmonary artery pulsatility index. *p < 0.01.

**FIGURE 3:**
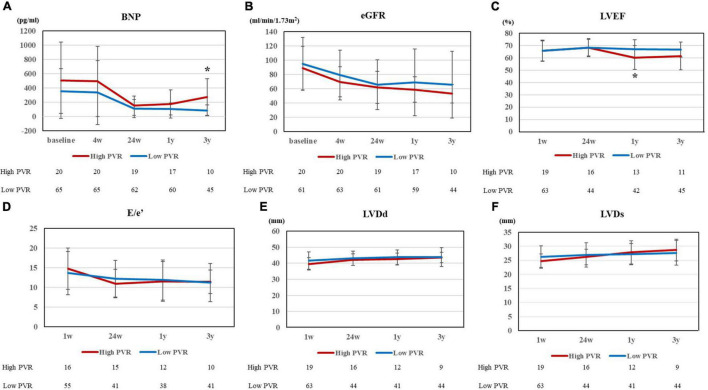
**(A)** BNP and **(B)** eGFR at 5 points (baseline, 4 weeks, 24 weeks, 1 year, and 3 years after HTx), **(C)** LVEF, **(D)** E/e’, **(E)** LVDd, and **(F)** LVDs at 4 points (1 week, 24 weeks, 1 year, and 3 years after HTx) for high and low PVR group. BNP, brain natriuretic peptide; eGFR, estimated glomerular filtration ratio; HTx, heart transplantation; LVEF, left ventricular ejection fraction; LVDd, left ventricular end diastolic dimension; LVDs, left ventricular end systolic dimension; and PVR, pulmonary vascular resistance. *p < 0.01.

Figure 2 shows the changes in clinical parameters after HTx. After HTx, PVR improved in both groups [high PVR group, 3.48 ± 0.64 WU (baseline) vs. 1.54 ± 0.53 WU (24 weeks after HTx), *p* < 0.0001; low PVR group, 1.65 ± 0.59 WU (baseline) vs. 1.32 ± 0.61 WU (24 weeks after HTx), *p* = 0.0016]. The PVR levels in both groups became comparable 1 year after HTx; however, the difference between each group became evident 2 years after HTx (Figure 2A). In contrast, the DPG and TPG levels were comparable in all times between two groups (Supplementary Figure 1). The differences in mPAP, mRAP, and mPCWP also tended to increase from 1 year after HTx and were all significantly higher in the high PVR group at 3 years after HTx [mPAP, 22.7 ± 9.0 mm Hg (high PVR group) vs. 15.4 ± 4.3 mm Hg (low PVR group), *p* = 0.0009; mRAP, 7.2 ± 3.6 mm Hg (high PVR group) vs. 4.1 ± 2.1 mm Hg (low PVR group), *p* = 0.0042; and mPCWP, 13.4 ± 4.5 mm Hg (high PVR group) vs. 8.8 ± 3.3 mm Hg (low PVR group), *p* = 0.0040] (Figures 2B–D). In the high PVR group, PAPi tended to decrease gradually in the chronic phase after HTx, which became significantly lower than that in the low PVR group at 3 years after HTx [2.51 ± 1.00 (high PVR group) vs. 5.21 ± 3.23 (low PVR group), *p* = 0.0033] (Figure 2F). BNP level was significantly higher in the high PVR group at 3 years after HTx, whereas eGFR was comparable in all times between the two groups (Figures 3A,B). Moreover, there was no significant difference in LVEF, LVDd/Ds and E/e’ and right ventricular function parameters between the two groups (Figures 3C–F and Supplementary Figure 2). By contrast, there was no significant difference in the presence of significant tricuspid regurgitation 3 years after HTx.

Table 4 shows the oral medications that were used 3 years after HTx. The number of patients using loop diuretics was significantly higher in the high PVR group (*p* = 0.016). These results suggested that increased burden on both left and right ventricles in patients of the high PVR group. In contrast, there were no significant differences in immunosuppressants, beta-blockers, ACE inhibitors, or MRAs between the two groups.

**TABLE 4:** Oral medication 3 years after HTx in two groups.

	High PVR (*n* = 10)	Low PVR (*n* = 45)	*P* value
**Medication 3 years after HTx**			
β-blocker, *n* (%)	9 (90)	35 (77.8)	0.35
ACE-I/ARB, *n* (%)	6 (60)	28 (62.2)	0.90
MRA, *n* (%)	2 (20)	6 (13.3)	0.60
Loop diuretics, *n* (%)	4 (40)	4 (6.7)	0.016*
Tacrolimus, *n* (%)	3 (30)	28 (62.2)	0.084
Cyclosporine, *n* (%)	7 (70)	17 (37.8)	0.084
Everolimus, *n* (%)	7 (70)	39 (86.7)	0.34

PVR, pulmonary vascular resistance; HTx, heart transplantation; ACE-I, angiotensin converting enzyme inhibitor; ARB, angiotensin receptor blocker; and MRA, mineralocorticoid receptor antagonist. *p < 0.05.

### Clinical Events After Heart Transplantation

**FIGURE 4:**
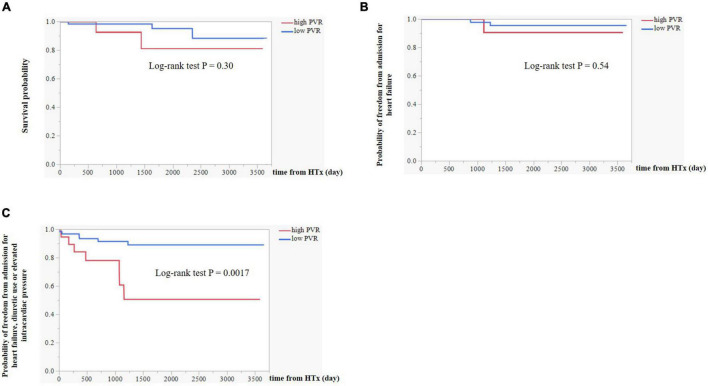
**(A)** Kaplan–Meier survival curve analysis between high PVR group and low PVR group. **(B)** Differences in event-free survival curves of admission for heart failure between high PVR group and low PVR group. **(C)** Differences in event-free survival curves of the composite event including admission for heart failure, diuretic use or elevated intracardiac pressure (mRAP ≥ 12 mm Hg or mPCWP ≥ 18 mm Hg) between high PVR group and low PVR group. PVR, pulmonary vascular resistance; mRAP, mean right atrial pressure; and mPCWP, mean pulmonary capillary wedge pressure.

In the Kaplan–Meier survival curve analysis, the presence of high PVR after LVAD implantation did not affect survival after HTx (*p* = 0.30, Figure 4A). During the follow-up period after HTx, one patient in the high PVR group was hospitalized for heart failure, while two patients in the low PVR group were hospitalized for heart failure after HTx (*p* = 0.54 Figure 4B). Subsequently, we examined the risk factors for the composite events of worsening heart failure, which included heart failure hospitalization, diuretic use for the treatment of congestion, and elevated intracardiac pressure. These were significantly more common in the high PVR group (*p* = 0.0017, Figure 4C). As shown in Table 5, residual PVR and donor age were important predictors of this combined event [(1) residual PVR, hazard ratio (HR) 4.9, 95% CI 1.6–14.7, *p* = 0.0058; (2) donor age, HR 6.8, 95% CI 2.2–21.0, *p* = 0.0008]. By performing multivariate analysis using both these factors and pre-HTx eGFR, residual PVR proved to be a significant predictor for this combined event (HR 6.5, 95% CI 2.0–21.6, *p* = 0.0023).

**TABLE 5:** Univariate and multivariate Cox proportional analysis of factors that determined the risk of the composite event of admission for heart failure, diuretic use or elevated intracardiac pressure (mRAP ≥ 12 mm Hg or mPCWP ≥ 18 mm Hg).

	Univariate analysis			Multivariate analysis		
	HR	95% CI	*P* value	HR	95% CI	*P* value
**Recipient factors**						
Residual PVR (PVR ≥ 3 WU, PVR < 3 WU)	4.9	1.6–14.7	0.0058**	6.5	2.0–21.6	0.0023**
Age (≥46 years, <46 years)	0.83	0.26–2.7	0.76			
Days from LVAD implantation to HTx (≥1,120 days, <1,120 days)	0.61	0.20–1.9	0.38			
BMI (≥25, <25)	1.8	0.49–6.5	0.40			
eGFR (<60 ml/min/1.73 m^2^, ≥60 ml/min/1.73 m^2^)	3.1	0.94–10.4	0.086*	4.6	1.3–16.5	0.031**
**Donor factors**						
Age (≥56 years, <56 years)	6.8	2.2–21.0	0.0008**	8.9	2.6–31.3	0.0004**
BMI (≥25, <25)	0.88	0.24–3.2	0.85			
Cardiac arrest	0.66	0.21–2.1	0.47			
**Donor-recipient relationship factors**						
Sex mismatch	1.8	0.54–5.8	0.36			
Donor to recipient BW mismatch < −20% or >30%	0.29	0.037–2.2	0.16			

HR, hazard ratio; CI, confidence interval; PVR, pulmonary vascular resistance; WU, wood unit; LVAD, left ventricular assist device; HTx, heart transplantation; BMI, body mass index; eGFR, estimated glomerular filtration rate; and BW, body weight. *p < 0.10, **p < 0.05.

## Discussion

In this study, we examined post-heart transplant hemodynamics and prognosis in patients stratified by the severity of PVR after LVAD implantation as BTT. The result was that residual high PVR under LVAD support corresponded to high right atrial→RAP and PCWP and low PAPi in the long term after HTx. As a result, patients with residual high PVR corresponded to high percentage of diuretic use. The novel point of this manuscript was a concise demonstration of the effect derived from residual high PVR during LVAD support on cardiovascular function after HTx, which had been not reported yet.

### Residual Pulmonary Vascular Resistance Increase After Left Ventricular Assist Device Implantation

There were some reports that high PVR before HTx has a detrimental effect after HTx (17–19). Chang et al. demonstrated mild to moderate preoperative pulmonary hypertension is associated with increased risk of developing early post-transplant pulmonary hypertension, which corresponded to early mortality after HTx. Conversely, there are also conflicting reports that high PVR does not affect the prognosis after HTx. Vakil et al. reported pretransplant pulmonary hypertension did not affect survival after HTx using UNOS registry, which included approximately 10% of patients with MCS support before HTx (20). Lindelow et al. reported that high PVR decreased after HTx and it did not have an impact on the survival after HTx (21). There were other similar results that pretransplant high PVR did not affect the survival after HTx (22–25). For explaining these conflicting results, the timing and patient condition of evaluating PVR seems to be critical for each result. Indeed, Shah et al. examined posttransplant survival between patients with high and low PVR, which was measured before mechanical support for bridging to HTx (24), whereas Drakos et al. used the value of PVR just before heart transplant (26). The characteristics of this study was that all patients were supported by LVAD before HTx and PVR was measured during LVAD support. Generally, the burden of pulmonary vasculature from left-sided heart failure is alleviated by LVAD support (8), so that the high PVR measured during LVAD support (“residual high PVR”), which was investigated in the current study, might reflect the increase in pulmonary vascular tone due to pathological remodeling, which indicates irreversible change. Indeed, in this study, the effect derived from the high PVR under LVAD support had a significant impact on the hemodynamics and status in heart failure in the long term even more than 2 years after HTx. Bollano et al. (27) demonstrated that patients with pulmonary hypertension (mPAP ≥ 23 mm Hg) 1 year after HTx had a significantly higher rate of death or retransplantation during the 5-year follow-up after HTx (*p* = 0.009). These results demonstrated the remaining pulmonary hypertension after HTx had continuously negative impact on the long-term clinical course after HTx. In the discussion about pulmonary vascular tone, the difficulty in accurately measuring pulmonary vascular tone abnormalities is noted. Indeed, the abnormality could not be detected by the value of PVR temporally after HTx in this study. It might be due to low sensitivity of PVR for the detection of pathological pulmonary vascular tone in normal hemodynamics, which is also the case in DPG and TPG. The more useful marker for the detection of pulmonary vascular tone should be developed in further study, which might lead to more concise elucidation between pulmonary vasculature and cardiac function. Lundgren et al. demonstrated the deranged PVR response during exercise despite normal PVR at rest after HTx (28). It might be difficult to represent the abnormalities in pulmonary vasculature by the hemodynamics at rest condition. Moreover, the exercise load test had been reported to evaluate pulmonary vascular dysfunction more concisely in pulmonary hypertension (29). A study on pulmonary vascular dysfunction in patients after HTx using exercise load test is warranted.

### Right Heart Dysfunction After Heart Transplantation

The result of this study revealed long-term right heart dysfunction after HTx might be derived from remaining high resistance of pulmonary artery before HTx. Multivariate analysis revealed that this factor affected late requirement of diuretics to the same extent with recipient renal function and donor factors, such as donor age. Generally, non-specific primary graft failure is considered the most important cause of right heart dysfunction early after HTx (12, 30). Primary graft failure is commonly precipitated by various pathways (31). Primary graft dysfunction become obvious in the left ventricle (19%), both ventricles (7%), and right ventricle (5%; 32). The determining factors for the development of primary graft failure were reported to be transplant operation associating factors, such as ischemic time. Conversely, there are some reports about the change in cardiac function of transplanted heart in the chronic phase (33). Goland et al. demonstrated the gradual impairment of both ventricles early after HTx, which was finely exemplified by tissue Doppler imaging on echocardiography (34). D’Andrea et al. demonstrated that the decrease in heart function in the right side was more obvious than that in the left side after HTx (35). This gradual decrease in right ventricle function might clarify the burden of pulmonary vasculature in this study. Compared with the low PVR group, PAPi, which is one parameter of right heart function, was significantly lower and RAP was significantly higher in the high PVR group at 3 years after HTx, suggesting that high PVR led to the burden of right ventricle. The consistent trend of high PVR in the high PVR group suggested sustained burden from pulmonary vasculature on the right ventricle led to the derangement of right heart, leading to graft dysfunction. Whal et al. demonstrated no difference in right ventricular function between patients with and without pretransplant pulmonary hypertension (36), which was different from our results. It might be due to different definitions of high PVR. Indeed, the definition of high PVR in the current study was measured under LVAD support, which corresponded to more severe pulmonary vascular dysfunction. In the current study, PCWP was also significantly higher in the high PVR group 3 years after HTx. There was no case of AMR, which is one of the factors that increase PCWP after HTx (37), in our study. In addition, the percentage of donor with high age (such as more than 60) was comparable in patients with high and low PVR groups, which suggested the difference in hemodynamics might not be derived from the characteristics of donor heart. A mediation analysis demonstrated the increase in PCWP 3 years after HTx was mainly derived from the increase in right sided pressures (Supplementary Figure 3) and it was suggested that the load of left side might be derived from right side burden. According to the impact of transplant vasculopathy, there was no significant difference in hemodynamic parameters in patients with and without obvious transplant vasculopathy (Supplementary Figure 4). However, we couldn’t perform concise evaluations about microvascular graft dysfunction and further studies investigating the association between right and left side burden of transplanted heart should be performed.

By Kaplan–Meier event-free survival curve, there was no significant difference in survival rate or admission due to heart failure after HTx between the high and low PVR groups. However, there were significantly more cases of loop diuretic use, and the BNP level was significantly increased in the high PVR group, both of which suggested the higher risk of heart failure in patients in the high PVR group after HTx. We analyzed the risk of worsening heart failure which was defined as heart failure hospitalization, diuretic use, and elevated intracardiac pressure. Indeed, the event of outpatient diuretic intensification as the sign of worsening heart failure in outpatient setting have been getting more attention (16, 38, 39), which was demonstrated to be comparable in the risk prognostication with heart failure hospitalization in patients with heart failure. However, the addition of diuretics and the increase in intracardiac pressure may be derived from renal dysfunction, and it is much difficult to distinguish the cause. Moreover, the significant decrease in PAPi corresponded to right ventricular dysfunction, which might critically limit exercise capacity (40). The increase in diuretic use or decrease in right ventricular function inevitably decrease the quality of life after HTx, which should be noted. However, there was no significant change in echocardiographic parameters in RV functions. We should investigate about the mechanistic insights including RV function of the findings in the future study.

### Study Limitations

First, besides the inherent limitation of a retrospective study, we examined small cases in a single center. We should further increase the study patients including different patient cohorts to verify the finding in this study. Second, patient selection bias can be mentioned. In this study, patients undergoing right heart catheterization after LVAD implantation were divided into two groups. We excluded cases in which different right heart catheterization protocol was adopted because of LVAD implantation in other institutions. Other severe cases, such as early death after cardiac transplantation and death during the perioperative period of the LVAD, were excluded. Moreover, 19 patients died during LVAD placement, of which 3 had high PVR and 14 had low PVR and 2 had missing data.

Third, the types of LVAD were not unified; extracorporeal LVAD, LVAD with implantable axial flow pumps, and LVAD with implantable centrifugal pumps were used. Baseline PVR could be affected by the different hemodynamics of various LVAD. Moreover, PVR after LVAD implantation was somewhat dependent on the characteristics derived from LVAD support, which might be changed in different mechanical supports, such as total artificial heart (23). In addition, the value of PVR might be affected by the addition of pulmonary vasodilatory agents such as phosphodiesterase 5 (PDE5) inhibitors. However, there was no significant difference in PDE5 inhibitors between two groups. Fourth, we checked PVR at one time point “1 month after LVAD implantation” during LVAD support so that PVR at the timing of HTx might be slightly different from that measured. Indeed, Gulati et al. demonstrated the change in PVR after LVAD implantation (41). They showed the highest percentage of high PVR (defined as PVR > 3 WU) in patients under LVAD 1 month after LVAD implantation (35%), which was gradually decreased later, such as 25% at 3 months, 20% at 6 months, and 15% at 24 months after LVAD implantation. The value of PAP and PVR continuously fluctuates during all the time after the LVAD implantation. Therefore, the appropriate timing for measurement of PVR after LVAD implantation is extremely difficult to decide. However, in this study, the exact value of PVR was not comparatively important but the identification of patients with residual high PVR was critical for the analysis. Therefore, the timing of PVR under LVAD support seems to be a somewhat low priority issue. In addition, there was no significant difference in the duration from LVAD implantation to HTx between groups. Fifth, we used composite endpoint of worsening heart failure, which might be subject to bias. However, the percentage of loop medication and the hemodynamic data supports the validity of the result of composite endpoint.

## Conclusion

Our study demonstrates that patients with residual high PVR under LVAD showed the increase of right and left atrial pressure in the chronic phase after HTx. The residual high PVR under LVAD implantation corresponded to the increase of diuretic use in the chronic phase after HTx.

## Data Availability Statement

The raw data supporting the conclusions of this article will be made available by the authors, without undue reservation.

## Ethics Statement

The studies involving human participants were reviewed and approved by the Institutional Review Board of the University of Tokyo Graduate School (assignment number: 2650). Written informed consent for participation was not required for this study in accordance with the national legislation and the institutional requirements.

## Author Contributions

NK: conceptualization, methodology, data curation, validation, and writing – original draft, review, and editing. EA: conceptualization, methodology, validation, data curation, and writing – review and editing. MH, MA, SS, OK, MO, and IK: supervision. MT, CB, JI, HY, AS, KN, YI, and KF: data curation. All authors contributed to the article and approved the submitted version.

## Conflict of Interest

EA belongs to the Department, endowed by NIPRO-Corp, Terumo-Corp., Senko-Medical-Instrument-Mfg., Century-Medical, Inc., ONO-pharmaceutical-Co., Ltd. Medtronic-JAPAN Co., Ltd, Nippon-Shinyaku Co., Ltd, Mochida Pharmaceutical Co.; Boehringer Ingelheim Pharmaceuticals Inc., Abiomed-Inc, AQuA-Inc, Fukuda-Denshi Co., Ltd, and Sun-Medical-Technology-Research Corp. The remaining authors declare that the research was conducted in the absence of any commercial or financial relationships that could be construed as a potential conflict of interest.

## Publisher’s Note

All claims expressed in this article are solely those of the authors and do not necessarily represent those of their affiliated organizations, or those of the publisher, the editors and the reviewers. Any product that may be evaluated in this article, or claim that may be made by its manufacturer, is not guaranteed or endorsed by the publisher.
