# Supplementary material for: Residual Pulmonary Vascular Resistance Increase Under Left Ventricular Assist Device Support Predicts Long-Term Cardiac Function After Heart Transplantation
Source: Front Cardiovasc Med. 2022 Jun 1;9:904350. doi: 10.3389/fcvm.2022.904350 (PMC9198244; doi:10.3389/fcvm.2022.904350)
Supplement: Supplementary file 1 [file Presentation_1.PPTX]

## Slide 1
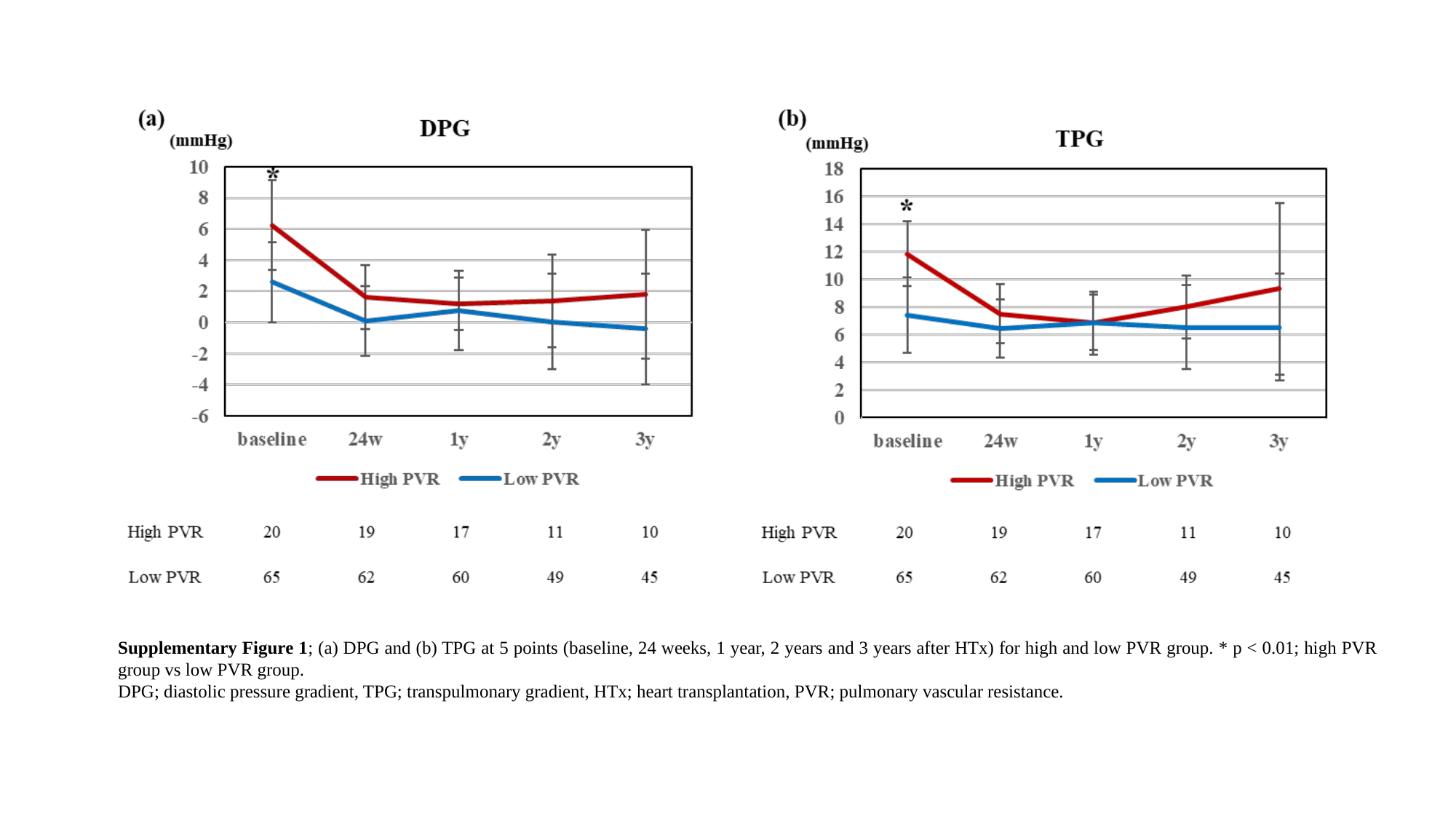

Supplementary Figure 1; (a) DPG and (b) TPG at 5 points (baseline, 24 weeks, 1 year, 2 years and 3 years after HTx) for high and low PVR group. * p < 0.01; high PVR group vs low PVR group.
DPG; diastolic pressure gradient, TPG; transpulmonary gradient, HTx; heart transplantation, PVR; pulmonary vascular resistance.

## Slide 2
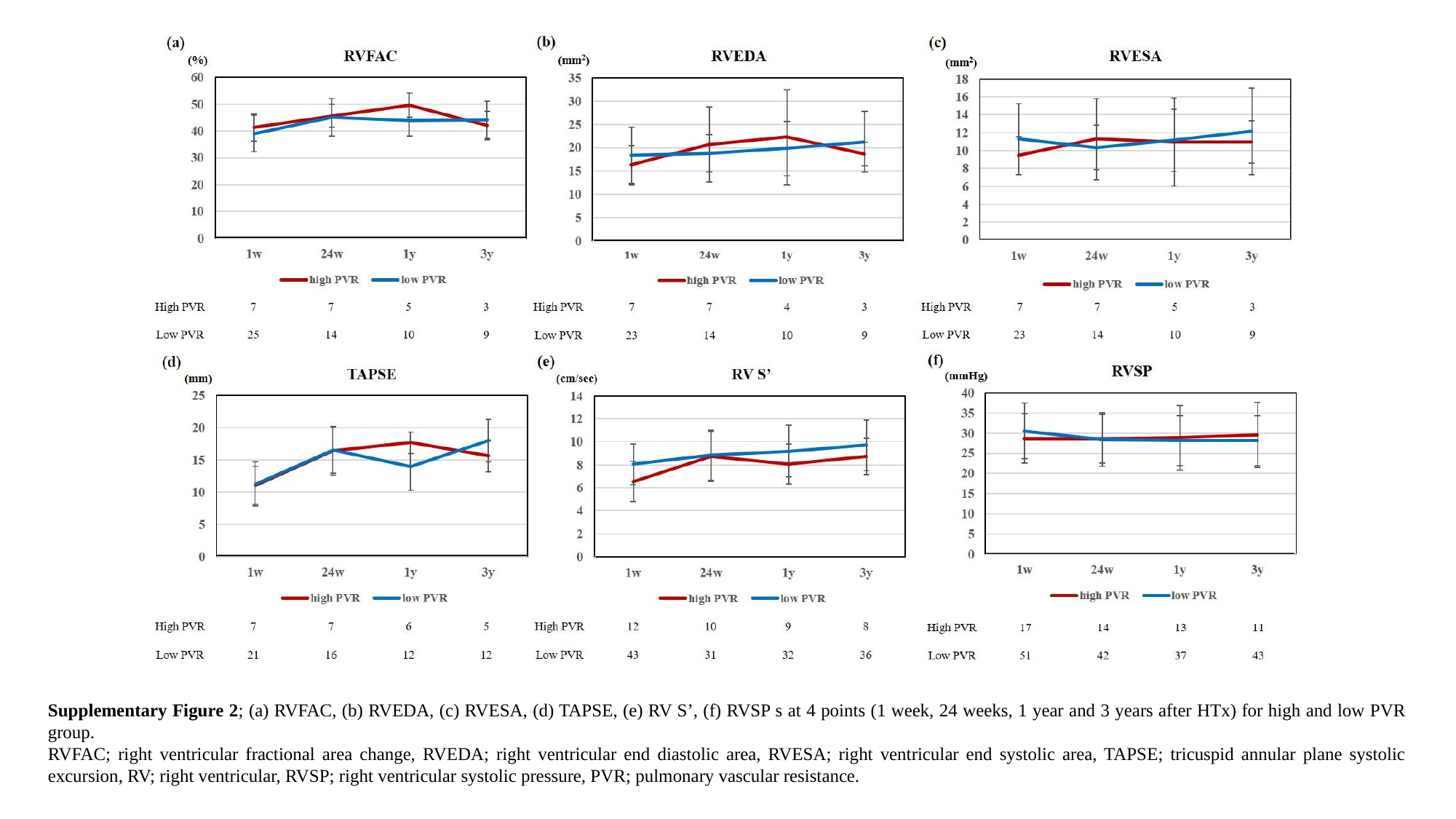

Supplementary Figure 2; (a) RVFAC, (b) RVEDA, (c) RVESA, (d) TAPSE, (e) RV S’, (f) RVSP s at 4 points (1 week, 24 weeks, 1 year and 3 years after HTx) for high and low PVR group.
RVFAC; right ventricular fractional area change, RVEDA; right ventricular end diastolic area, RVESA; right ventricular end systolic area, TAPSE; tricuspid annular plane systolic excursion, RV; right ventricular, RVSP; right ventricular systolic pressure, PVR; pulmonary vascular resistance.

## Slide 3
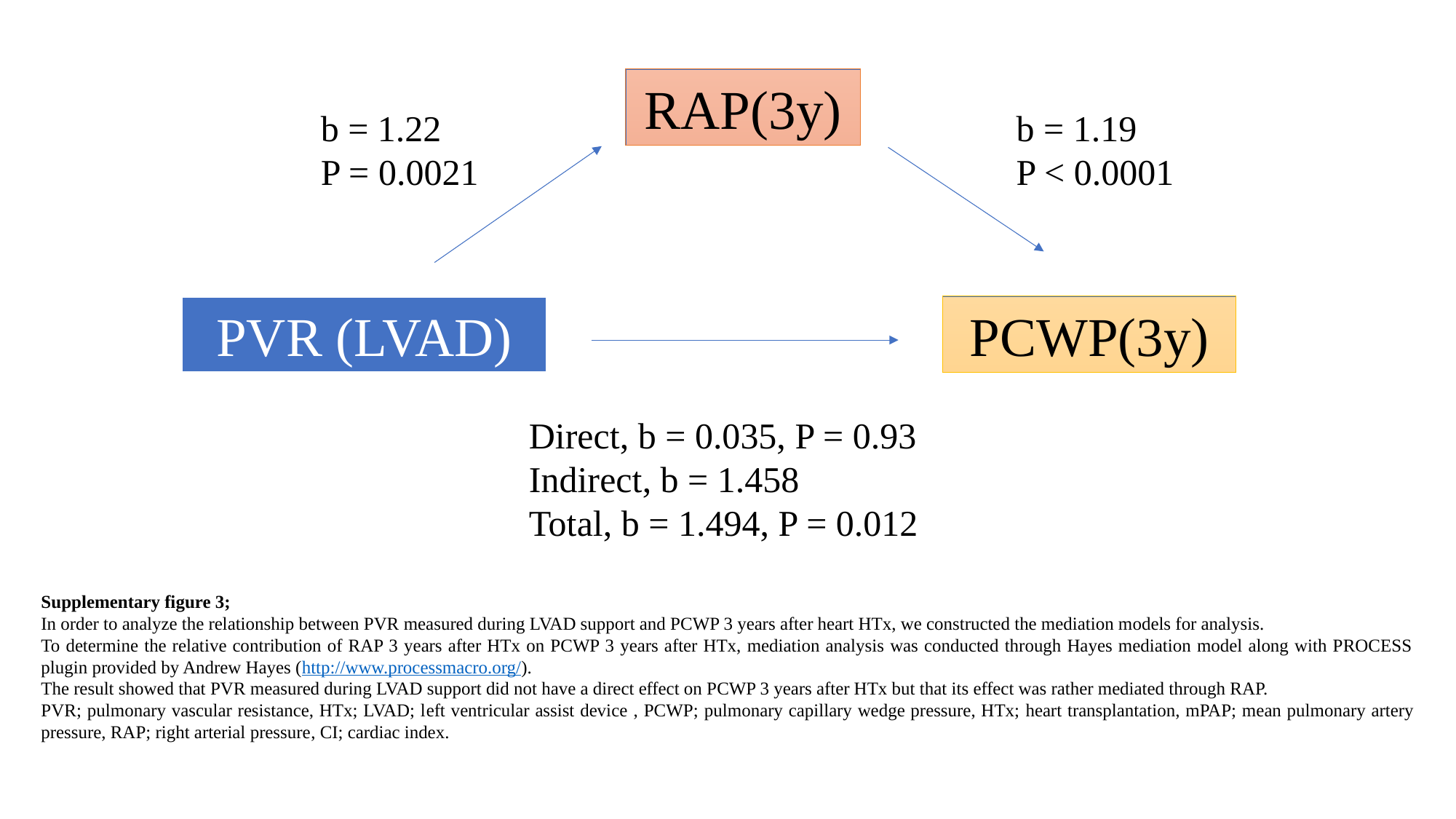

RAP(3y)
b = 1.22
P = 0.0021
b = 1.19
P < 0.0001
PCWP(3y)
PVR (LVAD)
Direct, b = 0.035, P = 0.93
Indirect, b = 1.458
Total, b = 1.494, P = 0.012
Supplementary figure 3;
In order to analyze the relationship between PVR measured during LVAD support and PCWP 3 years after heart HTx, we constructed the mediation models for analysis.
To determine the relative contribution of RAP 3 years after HTx on PCWP 3 years after HTx, mediation analysis was conducted through Hayes mediation model along with PROCESS plugin provided by Andrew Hayes (http://www.processmacro.org/).
The result showed that PVR measured during LVAD support did not have a direct effect on PCWP 3 years after HTx but that its effect was rather mediated through RAP.
PVR; pulmonary vascular resistance, HTx; LVAD; left ventricular assist device , PCWP; pulmonary capillary wedge pressure, HTx; heart transplantation, mPAP; mean pulmonary artery pressure, RAP; right arterial pressure, CI; cardiac index.

## Slide 4
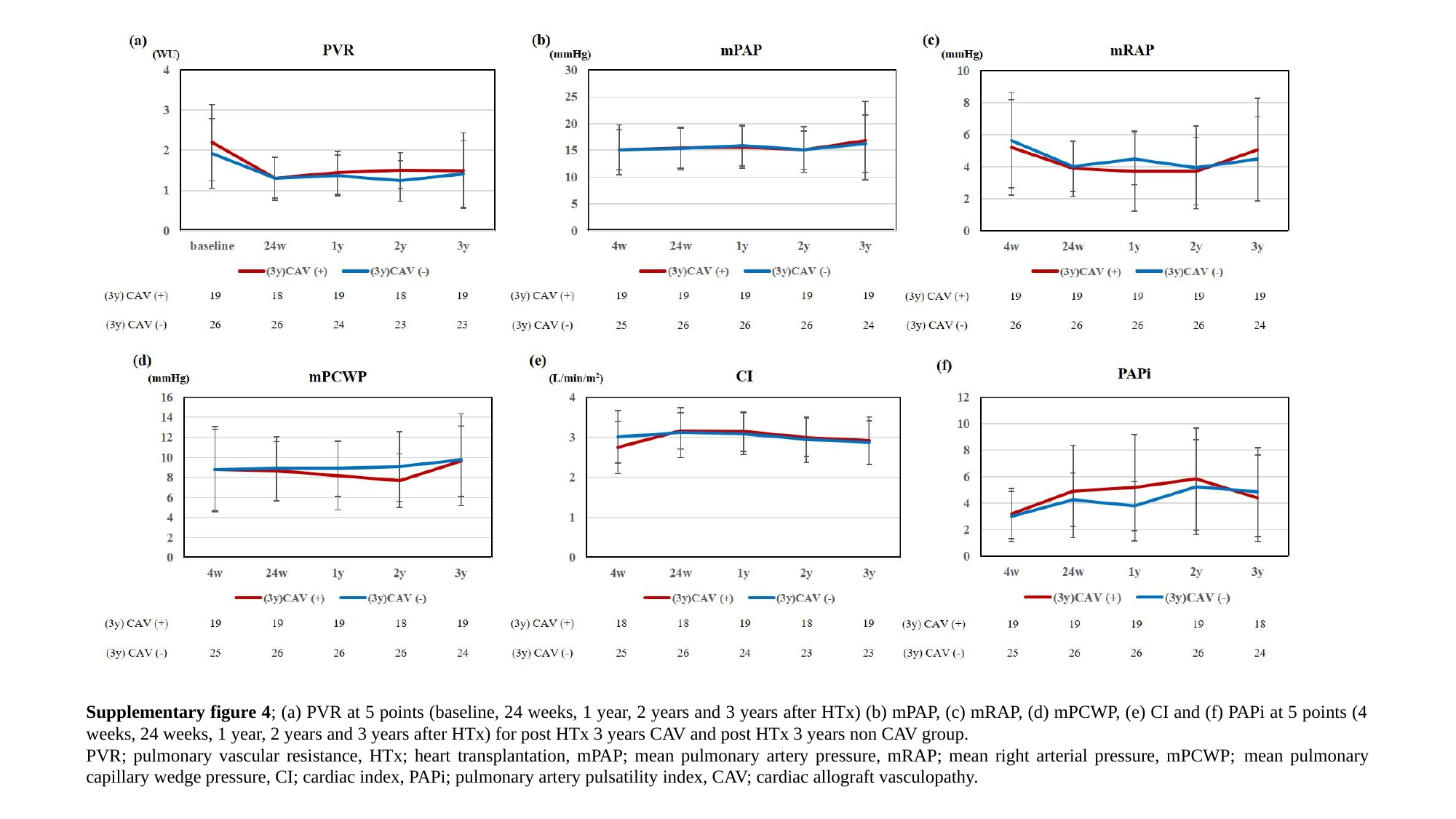

Supplementary figure 4; (a) PVR at 5 points (baseline, 24 weeks, 1 year, 2 years and 3 years after HTx) (b) mPAP, (c) mRAP, (d) mPCWP, (e) CI and (f) PAPi at 5 points (4 weeks, 24 weeks, 1 year, 2 years and 3 years after HTx) for post HTx 3 years CAV and post HTx 3 years non CAV group.
PVR; pulmonary vascular resistance, HTx; heart transplantation, mPAP; mean pulmonary artery pressure, mRAP; mean right arterial pressure, mPCWP; mean pulmonary capillary wedge pressure, CI; cardiac index, PAPi; pulmonary artery pulsatility index, CAV; cardiac allograft vasculopathy.
